# Epigenetic Erosion in Adult Stem Cells: Drivers and Passengers of Aging

**DOI:** 10.3390/cells7120237

**Published:** 2018-11-29

**Authors:** Christian Kosan, Florian H. Heidel, Maren Godmann, Holger Bierhoff

**Affiliations:** 1 Institute of Biochemistry and Biophysics, Center for Molecular Biomedicine (CMB), Friedrich Schiller University Jena, Hans-Knöll-Str. 2, 07745 Jena, Germany; christian.kosan@uni-jena.de; 2 Leibniz-Institute on Aging—Fritz Lipmann Institute (FLI), Beutenbergstrasse 11, 07745 Jena, Germany; florian.heidel@leibniz-fli.de; 3 Innere Medizin 2, Hämatologie und Onkologie, Universitätsklinikum Jena, 07747 Jena, Germany

**Keywords:** adult stem cells, spermatogonial stem cells, hematopoietic stem cells, muscle stem cells, self-renewal, differentiation, epigenetic regulation, tissue maintenance, aging

## Abstract

In complex organisms, stem cells are key for tissue maintenance and regeneration. Adult stem cells replenish continuously dividing tissues of the epithelial and connective types, whereas in non-growing muscle and nervous tissues, they are mainly activated upon injury or stress. In addition to replacing deteriorated cells, adult stem cells have to prevent their exhaustion by self-renewal. There is mounting evidence that both differentiation and self-renewal are impaired upon aging, leading to tissue degeneration and functional decline. Understanding the molecular pathways that become deregulate in old stem cells is crucial to counteract aging-associated tissue impairment. In this review, we focus on the epigenetic mechanisms governing the transition between quiescent and active states, as well as the decision between self-renewal and differentiation in three different stem cell types, i.e., spermatogonial stem cells, hematopoietic stem cells, and muscle stem cells. We discuss the epigenetic events that channel stem cell fate decisions, how this epigenetic regulation is altered with age, and how this can lead to tissue dysfunction and disease. Finally, we provide short prospects of strategies to preserve stem cell function and thus promote healthy aging.

## 1. Introduction

**Figure 1:**
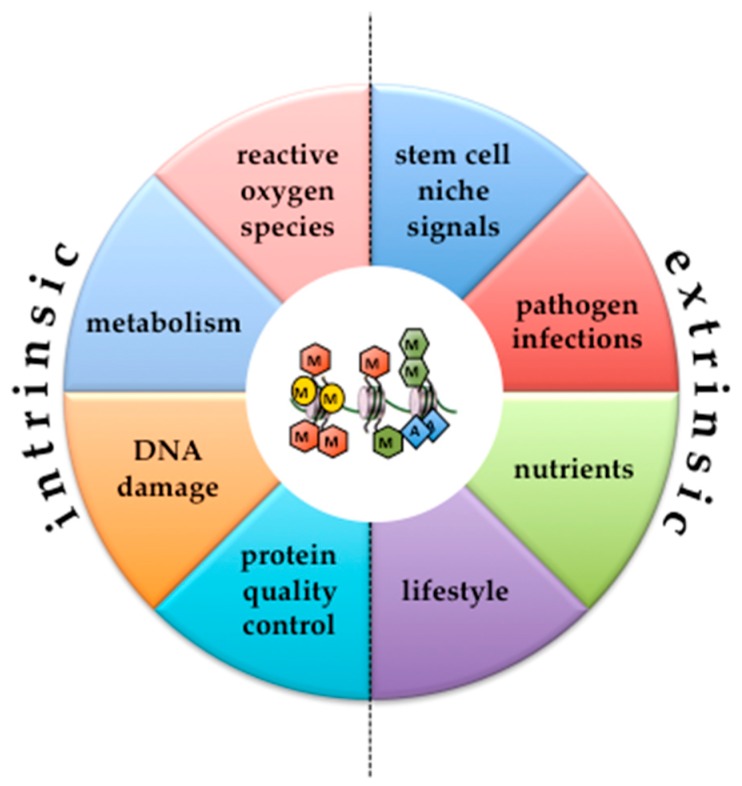
Intrinsic and extrinsic factors cause epigenetic erosion in adult stem cells. The diagram depicts major factors that contribute to stem cell aging and also affect the fidelity of epigenetic regulation (indicated in the middle by repressive histone methylation (red hexagons) or activating histone methylation (green hexagons); histone acetylation (blue diamonds) and DNA methylation (yellow circles). For further details please see text.

The statement “Every man desires to live long; but no man would be old,” made by the famous Irish writer Jonathan Swift in 1726 at the age of 60 describes in essence the motivation for the nowadays tremendous research efforts on healthy aging. A milestone in our understanding of the lifelong maintenance and regeneration of animal tissues was the discovery of hematopoietic stem cells, which paved the way for the identification of adult stem cells in virtually every tissue [1]. Given the pivotal role of adult stem cells in tissue homeostasis, their functional decline and exhaustion has been realized as a bona fide hallmark of aging [2]. Nevertheless, we are just beginning to understand what factors lead to impairment of stem cells with age, and how we can influence these factors to sustain the integrity of the different, and very heterogeneous stem cell compartments of an organism. Although stem cell aging has multifactorial causes, clearly epigenetics is at the core of most, if not all of them (Figure 1). Nowadays, there is ample evidence that extrinsic factors like nutrients, niche signals, and pathogen infections, as well as intrinsic factors like metabolism, DNA damage, and protein quality control, impact the epigenome of stem cells [3]. Consequently, aging-associated aberration of these extrinsic and intrinsic cues leads to epigenetic erosion, which might even accelerate itself, for instance, when epigenetic modifiers become mutated (see Section 3), or when histone genes become repressed (see Section 4).

An especially sensitive phase in stem cell development is the transition from quiescence to activation. Even stem cells in proliferative tissues of epithelial and connective types, including spermatogonial and hematopoietic stem cells, are mainly in a quiescent state to protect them from metabolic and replicative stress [4]. Upon activation, intricate epigenetic changes occur that, on the one hand, drive sufficient production of progenitor cells, but on the other hand make sure that stem cells do not lose their identity and the ability to self-renew. Perturbation of one or the other pathway causes either insufficient tissue regeneration or exhaustion of the stem cell pool. Growing evidence indicates that quiescent stem cells in old animals are indeed epigenetically impaired to properly respond to and process activation signals. In this review, we discuss recent work that connects fate decisions with epigenetic regulation and aging in spermatogonial stem cells (SSCs; Section 2), hematopoietic stem cells (HSCs; Section 3), and muscle stem cells (MuSCs; Section 4). While SSCs have a unique function as male germ cells that give rise to haploid spermatozoa, HSCs have to continuously replenish all types of blood cells. In contrast, MuSCs are mainly activated upon muscle damage. Thus, the compilation of these three heterogenous stem cell types provides a comprehensive overview of aging-associated epigenetic alterations at different developmental stages, the underlying mechanisms, and the consequences for tissue homeostasis. A common theme emerging is that from a cell’s huge epigenetic arsenal, especially DNA methylation regulated by DNA methyltransferases (DNMTs) and Ten-eleven Translocation (TET) dioxygenases, as well as particular histone modifications play a central role in stem cell aging. With regard to these histone modifications, the euchromatic histone H4 acetylation (H4ac) and MML1/2-mediated histone H3 lysine 4 trimethylation (H3K4me3) need to be well-balanced with the heterochromatic H3K27me3 mark, deposited by the polycomb repressive complex 2 (PRC2), and SUV420H2-mediated H4K20me3.

## 2. Spermatogonial Stem Cells

**Figure 2:**
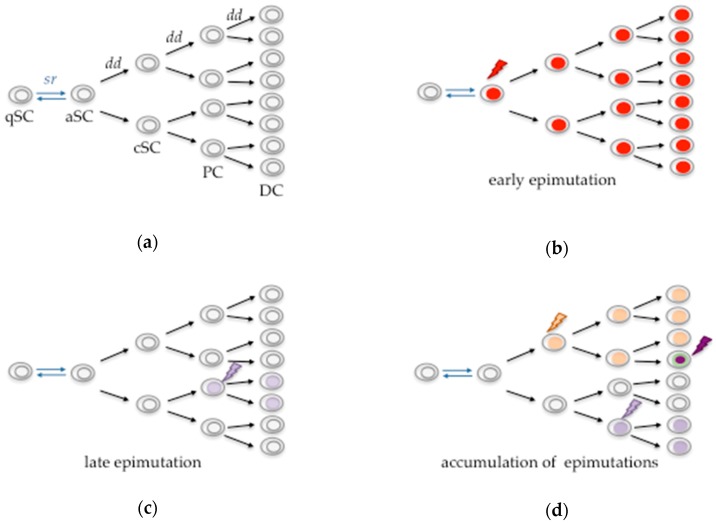
Transmission of epimutations during stem cell development. (**a**) Stages of stem cell development without epimutations. (**b**) Acquisition of an epimutation (indicated by a flash) at an early stage is transmitted to all subsequent progenitor and differentiated cells. (**c**) Occurrence of an epimutation at a late stage affects only a small population of differentiated cells. (**d**) Epimutations can accumulate during stem cell development. Abbreviations: quiescent stem cell (qSC); activated stem cell (aSC); committed stem cell (cSC); progenitor cell (PC); differentiated cell (DC); self-renewal (sr); differentiation division (dd).

SSCs are the male germline stem cell population of the postnatal testes that fuel spermatogenesis during the reproductive lifetime. SSCs either self-renew to maintain their own population or differentiate to eventually give rise to mature sperm [5,6]. The sequential transition of a diploid SSC into specialized haploid spermatozoa is a highly orchestrated, complex process and accompanied by unique epigenetic mechanisms, which enable a spatially and temporally regulated gene expression, proper progression through meiosis to generate haploid round spermatids, and the exchange of most of the histones by transition proteins and subsequently protamines to achieve compaction of the sperm head. Little is known about the effect of aging on spermatogenesis and on the health of future generation, as it was thought for a long time that a father’s sperm epigenome is insignificant for embryo development due to massive chromatin remodeling events in the early embryo [7,8]. Importantly, recent studies clearly demonstrate a correlation between epigenetic aberrations in male germ cells and sperm quality, fertility, and certain diseases in offspring, such as bipolar disorder, schizophrenia, or metabolic diseases, that cannot be explained by genetic alterations and where the female factor has been ruled out [9,10,11]. During a reproductive lifetime, epigenetic signatures (e.g., DNA methylation patterns, posttranslational histone modifications), and specific chromatin states (e.g., protamine-organized sperm nuclei) need to be re-established during each round of differentiation from SSC to sperm. Due to its intricacy, this process is error-prone and epimutations might occur at all steps of spermatogenesis. As SSCs undergo clonal amplification before they enter meiosis, the earlier aberrant epigenetic profiles are introduced, the more germ cells are affected and the higher are the chances of epimutations being transferred to the next generation (Figure 2). Thus, understanding how epimutations accumulate during parental aging and what the consequences are for fertility, reproductive outcome, and offspring health is an important question. In the next sections we focus on two key determinants of the chromatin structure: DNA methylation of CpG dinucleotides and histone modifications.

### 2.1. DNA Methylation Changes during Spermatogenesis

Comparison of whole genome bisulfite sequencing (WGBS) data obtained from pluripotent SSCs and terminal differentiated spermatozoa revealed almost no differences between both cell populations in mice and in humans, indicating that bulk DNA methylation profiles are maintained throughout postnatal spermatogenesis [12,13,14,15]. However, loci-specific gain or loss of DNA methylation has been observed during spermatogenesis and is key for its successful process. De novo DNA methylation patterns at various sites are created in a sequence-specific manner during early steps of murine spermatogenesis and completed by the end of the meiotic pachytene stage [15,16]. Interestingly, already established DNA methylation profiles might also change during spermatogenesis, as specific loci in committed SSCs lose DNA methylation upon differentiation at meiotic gene promoters for their upcoming expression in spermatocytes [14]. Various mouse models with genetic ablation of DNA methyltransferases convincingly prove that a failure in accurate, development-specific DNA methylation is often associated with impaired imprinting, reactivation of retrotransposons, meiotic defects, and impaired spermatogenesis [17,18,19,20]. Likewise, sperm DNA methylation profiles of infertile men have revealed that aberrations in DNA methylation at imprinted loci are associated with poor-quality sperm, impaired fertility, and reduced embryo quality during in vitro fertilization [21,22,23,24,25,26]. Intriguingly, several studies report an improvement of sperm quality and pregnancy rates when men were supplemented by vitamins, such as the methyl-group donor folic acid, and micronutrients, pointing towards the dynamic nature of the epigenome [27,28,29]. In addition to the dietary deficits, tobacco smoking and alcohol abuse lead to alterations of sperm DNA methylation profiles at imprinted loci and impair fertility [30,31,32,33]. Remarkably, De Castro Barbosa and colleagues also showed that epigenetic changes in the sperm epigenome induced by high fat diet of rats are paralleled by transgenerational inheritance of metabolic dysfunction throughout two generations [34]. It is conceivable that an unhealthy lifestyle might accelerate accumulation of epimutations in SSCs over time and thus might contribute to decreased male fertility with age.

### 2.2. Dynamic Regulation of Histone Modifications in SSC Development

In addition to DNA methylation, posttranslational histone modifications shape the epigenetic landscape in SSCs and are dynamic, though tightly controlled throughout spermatogenesis [35,36,37]. Noteworthy, in SSCs and early differentiating germ cells, repressive histone H3 trimethylation at lysine 9 and 27 (H3K9me3 and H3K27me3) and histone H4 trimethylation at lysine 20 (H4K20me3) display a low, but specific perinuclear distribution, pointing towards little heterochromatin in these cells [37]. In contrast, activating di- and trimethylation at lysine 4 of histone H3 (H3K4me2/3), is much more abundant in SSCs and progenitors. Strong H3K4me3 is predominantly found in early meiotic cells and in postmeiotic round and elongated spermatids, accompanying massive waves of transcription prior to meiotic chromatin compaction and histone-to-protamine exchange [35,38]. Global identification of histone modifications in various differentiating germ cell populations and mature spermatozoa revealed histone hyperacetylation from meiosis through specific postmeiotic stages as the most dynamic mark being mandatory for histone eviction [36,39]. After histone-to-protamine exchange, histone hyperacetylation sharply declines with only low residual acetylation of remaining histones in mature sperm. In accordance with these findings, a drastic reduction in acetylated histone H4 (H4ac), as well as a positional shift in the H3K9ac marks, was found in spermatids and spermatozoa of infertile men, suggesting an impaired histone-to-protamine exchange [40,41]. In this regard, it is interesting to note that the expression pattern of several histone deacetylases (HDACs) in SSCs is similar upon differentiation and aging [42], suggesting a loss of plasticity with age. Importantly, strong conservation of histone modifications in mature mouse and human spermatozoa was detected on histones H3 and H4, pointing towards their evolutionary function for retaining histones in spermatozoa [36]. Given that gene regulatory regions of embryonic developmental transcription factor genes lack DNA methylation signatures in SSCs, but are not expressed throughout spermatogenesis, their silencing is probably mediated by posttranslational histone modifications [12,43,44]. Interestingly, a bivalent epigenetic state, composed of activating H3K4me3 and repressive H3K27me3, is maintained during postnatal gametogenesis as it is found from SSCs to spermatozoa after histone-to-protamine exchange. Likewise, two other well-known markers of heterochromatin, H3K9me3 and H4K20me3, are transmitted by human spermatozoa to the zygote, uncovering propagation of heterochromatin as another example of paternal epigenetic inheritance [45]. The fact that intact histone signatures in spermatozoa matter for embryo development and offspring health in later life was clearly demonstrated by overexpressing histone demethylase 1 (Lsd1/Kdm1a) in murine SSCs and pre-meiotic spermatogonia. The resulting aberrant histone demethylation led to offspring with severe health defects [46].

Altogether, these examples demonstrate that dynamic, though highly coordinated, epigenetic processes in paternal germ cells are vital for fertility, embryogenesis, and health of future generations. Noteworthy, aging of sperm, and thus also indirectly of SSCs, can be determined using an epigenetic clock, i.e., DNA methylation patterns [47]. The questions regarding how epigenetic alterations with age affect SSC function, and how this process is influenced by environmental and lifestyle conditions, await further research.

## 3. Hematopoietic Stem Cells

Hematopoietic stem cells (HSCs) have the ability of self-renewal and produce cells of all blood lineages throughout life. During aging, HSCs lose their ability to self-renew and show a change in lineage contribution. This is associated with strong transcriptional changes and modification of the epigenetic landscape [48]. For instance, genes that are associated with the self-renewal capacity of HSCs can be inactivated via a stepwise increase in DNA methylation during aging [48]. How these epigenetic alterations are regulated is still a matter of ongoing research. Growing evidence indicates the contribution of several extrinsic factors like inflammation, as well as intrinsic mechanisms such as DNA damage or deregulation of epigenetic modifiers. These changes may eventually lead to epigenetic erosion and change the stem cell potential of aged HSCs progressively. Understanding these epigenetic changes will provide new strategies to improve HSC function during aging.

### 3.1. Impact of DNA Damage and Inflammation on the HSC Epigenome

Genotoxic stress can cause genetic alterations and cancer development during aging. HSCs are mainly quiescent to reduce the amount of DNA damage induced by intracellular reactive oxygen species (ROS) [49]. In fact, ROS are the major source of genotoxic stress in HSCs and more differentiated progenitors, and induce proliferation and activation of HSCs [50]. However, quiescent HSCs show reduced intracellular ROS levels, and this is dependent on Forkhead transcription factors [50]. Studies from mouse models showed that an inefficient DNA damage repair leads to accumulation of damage during aging, resulting in hematopoietic malfunction or even cancer development [51,52,53]. How HSCs, in particular in a quiescent state, respond to DNA damage is not fully understood yet. DNA damage can be a direct consequence of HSC activation by extrinsic stimuli. Repeated exposure to stressors, such as infection or blood loss, leads to exhaustion of HSCs [54]. Mohrin and colleagues showed that the exceptional radiation-resistance of quiescent HSCs is mediated by the DNA damage-recognizing kinase “Ataxia telangiectasia mutated” (ATM) [55]. On the other hand, quiescent HSCs mainly use the error-prone non-homologous-end-joining (NHEJ)-mediated DNA repair, while activated and proliferating HSCs use homologous recombination (HR), which decreases the risk of acquiring additional mutations [55]. DNA damage in HSCs induces strong up-regulation of proapoptotic genes (i.e., *bax*, *nova*, and *puma*) as well as *p21*, but no elimination by p53-mediated apoptosis. In contrast, more differentiated progenitors are mainly eliminated by induction of apoptosis [55]. Apoptosis-resistance of HSCs could be attributed to a high expression of pro-survival factors, which preserve the HSC pool, but also foster the accumulation of DNA lesions during aging. Rather than mediating apoptosis in HSCs, p53 regulates self-renewal capacity [56]. Interestingly, p53-deficient HSCs lose their ability to self-renew and accumulate mutations in serial transplantations even when anti-apoptotic signals were provided [56]. These findings clearly show that HSCs have unique ways to respond to genotoxic stress. How these cells accumulate mutations over time, but maintain cellular homeostasis, remains elusive.

In addition to genotoxic stress caused mainly by ROS, HSCs are exposed to other cellular stressors such as inflammation. Organismal aging is usually associated with chronic inflammation induced by anemia, immunosenescence, and thrombocytosis [57]. Furthermore, senescent cells display a specific senescence-associated secretory phenotype (SASP) and their increase with age leads to elevated levels of pro-inflammatory cytokines such as Interleukin 1 and 6 (IL-1 and IL-6), and tumor necrosis factor (TNF) [58]. HSCs maintain homeostasis of the hematopoietic system under steady state conditions by producing all types of blood cells. Interestingly, activation of HSCs by different stressors, such as anemia, induces a bias in the production of blood progenitors. This HSC response can be triggered by systemic inflammation leading to depletion of immune cells [59]. However, recent studies showed that HSCs also respond directly to both acute and chronic infections [60,61,62,63]. How this intrinsic response is mediated by epigenetic changes has not been shown in much detail, but with respect to the findings of trained innate immunity, this might be regulated in a similar way [64]. The stimulation of HSCs via infection and inflammation may also be mediated by changes in the immediate microenvironment. A range of studies indicated that HSC fate decisions are strongly influenced by cell intrinsic and extrinsic effects such as cells or factors of the microenvironment and the HSC niche [65,66,67]. The inflammatory stimuli trigger epigenetic changes in histone modifications and DNA methylation to adapt gene expression programs in HSCs [68,69]. Chronic inflammation might reinforce these changes and cause long term effects that contribute to aging and loss of functionality of the HSC pool over time. In line with this notion, it has recently been shown that various stressors can induce different epigenetic changes to drive independent clonal behavior [70].

### 3.2. Epigenetic Erosion of Hematopoietic Stem Cells and Clonal Aberration with Age

Aging of the human hematopoietic system is accompanied by various alterations of the epigenome including global DNA-methylation, nucleosome composition, chromatin-associated RNA, and protein expression, as well as post-translational histone tail modifications such as acetylation and methylation [3,71,72,73,74,75]. Changes in the global human hematopoietic DNA methylation profile reflect the biological age of the individual and can be tracked using specific CpG-sites [73,74,75]. Moreover, recent reports indicate age-related changes in heterochromatin leading to deregulation of non-coding RNA expression in animal models that may also apply to the hematopoietic system [76]. Other studies on human hematopoiesis indicate remarkable differences in cell-type- and hematopoietic-lineage-specific chromatin modification patterns during aging. Comparison of younger and older individuals has shown that aging is also associated with increased heterogeneity between individuals and elevated cell-to-cell variability in chromatin modifications [72]. The mechanisms of theses aging-associated changes in hematopoietic stem cells and their functional consequences are still not completely understood.

One major aspect that may explain epigenetic changes during aging is the development of clonal aberrations in the hematopoietic system of older individuals that have been recently discovered using whole exome sequencing of peripheral blood cells [77,78]. Notably, epigenetic modifiers were identified among the genes with the highest recurrence within the aging human hematopoietic system. Detectable somatic mutations were rare in persons younger than 40 years of age, but rose to more than 10% in persons over the age of 65 and up to 19.5% over the age of 90 [77,78,79]. The frequency of somatic mutations in the aging hematopoietic system, however, may still be underestimated due to the following aspects: (i) the published analyses are based on exome sequencing, which excludes identification of relevant mutational events in the non-coding (inter- or intragenic) regions, (ii) clonal aberrations may be cell-type-specific and therefore misrepresented in the periphery depending on the cell composition, and (iii) relevant mutations may arise in the stem and progenitor compartment of the bone marrow and therefore, depending on their functional impact, be underrepresented in mature blood cells. Very recent data support this aspect by describing a role for inflammatory signals in promoting myeloid differentiation of hematopoietic stem and progenitor cells (HPSCs) that are significantly influenced by the presence of cohesin mutations [80]. Here, cohesin mutations led to increased resistance to differentiation-inducing inflammatory stimuli. Linking cohesin with myeloid differentiation may help to explain the low prevalence of cohesin mutations in the peripheral blood of older individuals as compared to hematologic neoplasms.

### 3.3. Link between Aging-Induced Epimutations and Hematologic Cancer Formation

Mutations within the coding region of three epigenetic regulator genes—DNMT3A, TET2, and ASXL1—contribute to the majority of mutational events detectable in the peripheral blood during hematopoietic aging. These mutations may occur in otherwise healthy individuals with no apparent pathologies of blood production and maturation [81]. The presence of these aging-associated mutations, however, was associated with an increased risk for hematopoietic cancers [77,78], but also for cardiovascular disease and overall mortality [78,82]. These findings indicate that clonal mutations may affect the aging organism in several ways: DNMT3A and TET2 are responsible for de novo DNA methylation and demethylation, respectively, and will thereby influence the hematopoietic epigenome. Moreover, clonal hematopoiesis may result in the induction of inflammatory signaling, as demonstrated in murine models of TET2- or JAK2-mutated blood cells that reflect the increased risk for cardiovascular disease and inflammatory conditions in the aging human population. Inflammatory signals may themselves induce epigenetic changes. So far, it is not well understood how these epigenetic modifier mutations are connected to the impact of the inflammatory component and of epigenomic changes during aging independent of clonal aberrations. One example in this regard is the decline in human blood cell levels of 5-hydroxymethylcytosine (5hmC) during aging, which appears to be multifactorial and combines TET2-dependent and -independent aspects. While individuals with TET2 mutations have significantly lower 5hmC levels, persons harboring DNMT3A mutations do not [83]. However, TET2-mutations identified in this study could not account for the decline in 5hmC levels with age alone, as older individuals without clonal hematopoiesis also had lower 5hmC levels compared to their younger comparators. Otherwise, these somatic mutations may result in epigenetic changes that influence the clonal dominance of hematopoietic stem and progenitor cells. DNMT3A mutations, that account for the most frequent genetic aberrations in the aging hematopoietic system, can also be detected in myeloid neoplasia such as myelodysplastic syndrome (MDS) [84], supporting the concept that the underlying clonal architecture represents a fertile soil for development of hematopoietic cancers. While on one hand, the transformation rate from clonal hematopoiesis to myeloid neoplasms is below 1% per year [81], on the other hand, the occurrence of these mutations as early events in MDS clearly underlines their role in epigenetic erosion of hematopoietic stem cells and predisposition to malignant transformation. Their prognostic value in MDS, however, is still a matter of current debate: while early studies suggested impaired overall survival and increased transformation to acute myeloid leukemia (AML) [85,86], more recent investigations failed to confirm such a prognostic impact [87,88]. In acute myeloid leukemia the rate of DNMT3A mutations is even higher and is detected in up to 20% of patients [89]. Interestingly, cells harboring DNMT3A mutations can be frequently detected in patients with AML with long-lasting complete remission [90]. Several groups have reported that the persistence of DNMT3A mutations in the absence of disease is not associated with dismal clinical outcomes [91,92]. As a consequence, presence of DNMT3A mutations in patients with no evidence of residual leukemia may be considered as persistent preleukemic clones rather than as leukemia (stem) cells, and their use as markers for molecular detection of minimal residual disease has been argued against [93,94]. Although these mutations may therefore not act as primary drivers of myeloid neoplasms that predict prognosis or relapse, they will affect and modify the epigenetic landscape [95,96] of aged hematopoietic cells, which facilitates cooperation with other oncogenic mutations [96,97].

### 3.4. Therapeutic Implications of HSC Aging

From a therapeutic standpoint, mutations in epigenetic modifier genes may offer therapeutic vulnerabilities that suggest the use of hypomethylating agents (HMAs), such as 5-azacytidine or decitabine. HMAs have become standard therapeutics for AML and MDS patients [98,99]. These compounds aim to inhibit DNMTs and restore normal methylation levels at relevant promoter regions [100]. Analyses on AML cells harboring DNMT3A mutations revealed that despite global DNA hypomethylation specific regions of the genome are instead hypermethylated, also justifying the usage of HMAs in this context to restore normal methylation levels at these specific sites. On the other hand, in regions with low methylation levels, HMAs may further reduce global DNA methylation and lead to the synthetically lethal phenotype observed during clinical treatment [101,102]. Taken together, HMAs may counteract the consequences of epigenetic modifier mutations, which would be supported by the observation that DNMT3A-mutated patients seem to respond better to HMA therapy [103]. Finally, recent reports have shown marked overexpression of the histone H3K79 methyltransferase DOT1L in DNMT3A-deficient murine HSCs, resulting in increased H3K79 methylation. Pharmacologic inhibition of DOT1L in murine and human DNMT3A-mutated AML cells resulted in reduced proliferation, colony formation, and induction of cell death, suggesting that DOT1L could be an immediately actionable therapeutic target [104].

Aging-associated decline of HSC self-renewal, lineage potential, and homing capacity limit their use in hematopoietic stem cell transplantation [105]. This loss of regenerative capacity may eventually result in selection of an unrelated younger donor instead of related donors (siblings) of higher age. The seminal finding that transcription factors can revert adult somatic cells into pluripotent iPS cells [106] makes it tempting to speculate on rejuvenation of aged HSCs depending on their epigenetic landscape. In line with this notion is the striking anti-aging effect that can be achieved by transient expression of the reprogramming “Yamanaka” factors in old or progeric mice [107].

## 4. Muscle Stem Cells

**Figure 3:**
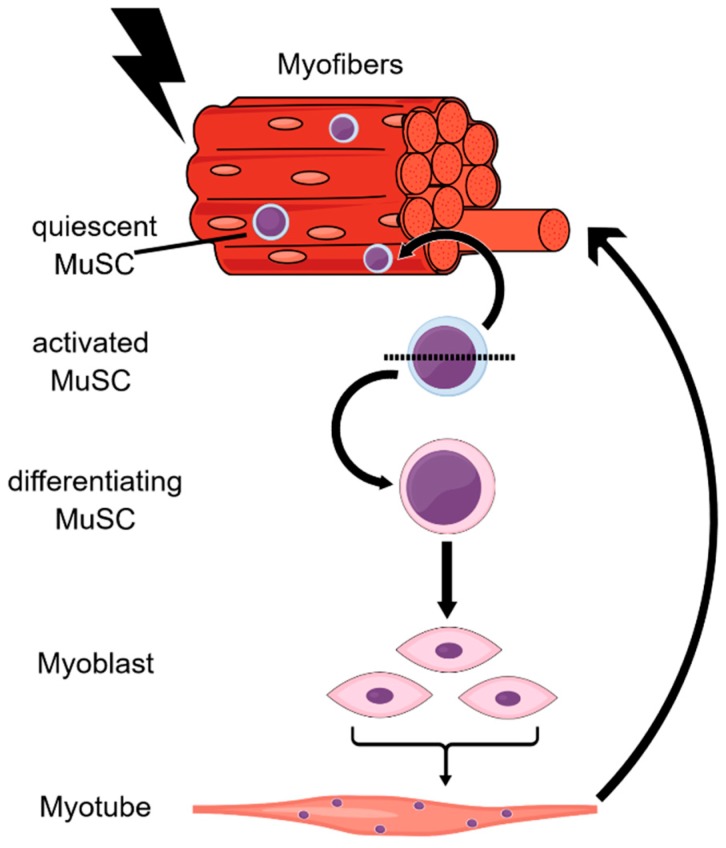
Scheme of the myogenic program. In response to muscle damage, quiescent MuSCs in the periphery of the myofibers become activated and enter the cell cycle. The daughter cells either self-renew into MuSCs, or they differentiate into myoblasts. These progenitor cells proliferate further until they eventually terminally differentiate and fuse with each other to form a myotube.

Skeletal muscle accounts for 30–40% of the body weight of adult humans [108]. Muscle tissue has a slow turnover under normal conditions, but has the formidable capacity to regenerate upon microtrauma induced by extensive exercise, or upon larger injuries. Both sporadic replenishment and lesion-induced regeneration depend on muscle stem cells (MuSCs) that are also termed satellite cells owing to their peripheral location underneath the myofiber basal lamina [109]. In adult muscles, 2–7% of nuclei at the basal lamina are MuSCs and most of them are in a quiescent state [110]. However, upon activation, MuSCs enter the cell cycle and produce committed progenitor cells, i.e., myoblasts, that amplify and eventually differentiate into fused myotubes (Figure 3). In addition, MuSCs have to self-renew to avoid exhaustion of the stem cell pool. The fate decisions of MuSCs and their daughter cells are highly regulated according to extrinsic signals from the stem cell niche, as well as intrinsic pathways, that together shape specific epigenetic signatures to facilitate differential transcription factor binding and gene expression [110,111].

Despite the muscles’ lifelong ability to regenerate, aging is associated with progressive muscle wasting, a process referred to as sarcopenia [112]. While sarcopenia has multifactorial causes, e.g., muscle denervation, impaired mobility, and reduced nutritional intake, there is also a strong contribution from the decline in MuSC numbers and function [113]. Thus, understanding the molecular mechanisms that perturb MuSCs self-renewal and regenerative potential with age is a prerequisite to counteract sarcopenia and foster healthy aging [114]. In this section, we summarize the epigenetic control of faithful MuSC development and will highlight the aberrant changes upon aging. A brief summary is given in Table 1:

### 4.1. Impact of H3K4me3 and H3K27me3 Marks on MuSC Activation

MuSCs fate decisions rely on myogenic regulatory factors (MRFs) that are expressed in a stage-specific manner [110,115]. The central factor conferring MuSCs identity is the paired box protein 7 (PAX7), whose activity is regulated via CARM1-mediated arginine methylation [116]. Whereas quiescent MuSCs (qMuSCs) harbor mainly unmodified PAX7, methylated PAX7 in activated MuSCs (aMuSCs) interacts with the MLL1/2-containing trithorax complex. Thereby, MLL1/2 is recruited to the *Myf5* promoter and activates expression of this MRF by depositing euchromatic histone H3 lysine 4 trimethylation (H3K4me3). Co-occurence of PAX7 and MYF5 is one of the first steps of myogenic commitment; however, other epigenetic changes accompany MuSC activation. In contrast to the acquirement of H3K4me3 at *Myf5* promoter in aMuSCs, this modification is already abundant in qMuSCs and marks about 50% of annotated gene promoters, including roughly 2000 bivalent promoters at which H3K4me3 co-exists with repressive H3K27me3 [117]. A major chromatin change upon activation is a strong increase in H3K27me3, which corresponds to the transcriptional up-regulation of the respective histone methyltransferase EZH2 belonging to the polycomb repressive complex 2 (PRC2) [117]. As the H3K27me3 gain occurs not only in gene bodies and intergenic regions, but also in H3K4me3-marked promoter regions, aMuSCs have higher levels of bivalent domains than qMuSCs.

### 4.2. Aberrant Regulation of H3K4me3 and H3K27me3 in MuSC Aging

Interestingly, H3K27me3 is markedly increase in aged qMuSCs, including both sites that already harbor the mark as well as sites that lack H3K27me3 in young qMuSCs [117]. The latter fraction includes many histone genes that in turn become down-regulated. Given that perturbed histone biosynthesis was found in replicative aging of cultured cells and is linked to DNA damage [118], H3K27me3-mediated silencing of histone genes in old qMuSCs is likely to contribute to epigenetic erosion. However, as the expression levels of EZH2 or H3K27me3-demethylases are not altered with age [117], the underlying mechanism remains elusive. Concomitant with the increase in H3K27 trimethylation in old qMuSCs, the intensity, but not the distribution, of the H3K4me3 mark was modestly decreased upon aging [117]. A striking exception from this trend has recently been shown for several genes encoding cell cycle inhibitors, as well as for the *Hoxa9* gene [119,120]. The increase of H3K4me3 up-regulates the cell cycle inhibitor genes, thus reducing the proliferative capacity of old MuSCs [119]. Moreover, in qMuSCs, *Hoxa9* together with other adjacent *Hoxa* genes is marked by H3K4me3, while the 5′ and 3′ ends of the *Hoxa* cluster harbor bivalent chromatin [117]. Stress-induced activation of qMuSCs triggers additional H3K4me3 deposition at *Hoxa9*; however, this epigenetic response is strongly increased with age [120]. As a consequence, *Hoxa9* is aberrantly expressed and induces signaling pathways that adversely affect MuSC function [120]. Given that these aging-associated deficits can be ameliorated by direct knockdown of *Hoxa9*, but also by pharmacological inhibition of the histone H3K4 trimethylating enzyme MLL1 [120], this study opens up new possibilities to improve the regenerative capacity of old MuSCs.

### 4.3. H4K20 Methylation Marks Play a Pivotal Role in Establishing MuSC Quiescence

A recent study has shown that methylation of histone H4K20 plays an important role in the maintenance of MuSC quiescence [121]. Di- and trimethylation states of H4K20 are mediated by SUV420H1 and SUV420H2, respectively, and are both associated with heterochromatin formation [122]. MuSCs deficient in SUV420H1, and thus H4K20me2, display a strong reduction in facultative heterochromatin, leading to aberrant activation of the *MyoD* gene [121]. Similar to MYF5, MYOD is a key MRF whose expression commits aMuSCs to the myogenic program [110,115]. Thus, loss of H4K20me2 interferes with MuSC quiescence, causing depletion of the stem cell pool in repeatedly injured muscles. Although H4K20me2 has a crucial role in qMuSCs, the levels do not change upon activation. In contrast, global levels of SUV420H2-mediated H4K20me3 are high in qMuSCs, but virtually undetectable in aMuSCs [121]. This observation complements previous findings that H4K20me3 is generally elevated upon quiescence, including terminal differentiated C2C12 myotubes [123]. Consequently, H4K20me3 is dynamically regulated during myogenesis, with high levels in qMuSCs, transient depletion in aMuSCs and myoblasts, and final restoration in myotubes.

### 4.4. Link between Epigenetic Regulation, Metabolism, and Muscle Aging

While H4K20me3 confers a repressive chromatin state, acetylation of the neighboring lysine- residue 16 (H4K16ac) is an activating epigenetic mark [124]. H4K16ac is the preferred histone substrate of the nicotinamid adenine dinucleotide (NAD^+)^-dependent histone deacetylase (HDAC) sirtuin 1 (SIRT1) [125]. Given that NAD^+^ is a metabolite of the mitochondrial adenosine triphosphate (ATP) production via oxidative phosphorylation, it transmits metabolic cues to chromatin by licensing SIRT1 activity. Notably, MuSC activation has been shown to be accompanied by a metabolic switch from oxidative phosphorylation to glycolysis, leading to decreased NAD^+^ levels, SIRT1 inactivation and ultimately to elevated H4K16 acetylation [126]. Increased H4K16ac levels derepress genes, including *MyoD*, that drive myogenic commitment, and mimicking this effect by SIRT1 ablation leads to premature MuSC differentiation [126]. These results together with data showing impairment of oxidative phosphorylation with age [127] provide one possible mechanism for aging-associated exhaustion of MuSC. Counteracting the resulting decline in regenerative capacity simply by diet-mediated improvement of oxidative phosphorylation is tantalizing. Indeed, short-term (12 weeks) caloric restriction in aged mice induces expression of SIRT1 and a shift towards oxidative metabolism, and, strikingly, increases the frequency and functionality of MuSCs [128]. Taken together, the direct link between metabolism and epigenetic regulation provides a promising opportunity to use nutritional interventions for counteracting aging-dependent stem cell impairment, and this beneficial effect may be even potentiated by exercise training [129]. In contrast to promoting SIRT1 activity, the Zn^+^-dependent class I and II HDACs require inhibition for a beneficial effect on muscle maintenance. In myoblasts, these HDACs target the promoters of the *MyoD* and *Fst* genes, the latter encoding Follistatin, an antagonist of the muscle growth inhibitor Myostatin [130]. Thus, HDAC inhibitors antagonize hypoactylation of histones at the *MyoD* and *Fst* promoters, causing increased expression. Moreover, HDAC inhibition conserves the acetylation of MYOD, which is required for its myogenic activity [131]. These epigenetic effects mediated by HDAC inhibitors have been shown to promote muscle regeneration in a mouse model of Duchenne muscular dystrophy, as well as to sustain muscle mass in aged mice [132,133]. Future studies will have to show whether the treatment with HDAC inhibitors is an effective measure to delay, or even to prevent, sarcopenia in elderly humans.

## 5. Conclusions

The lifespan of humans is rapidly increasing worldwide. According to the United Nations, people aged 60 or above are the fastest growing fraction of the world population, which is estimated to account for approximately 25% in the year 2050 (UN World Population Prospects; The 2017 Revision). This tremendous challenge for our social security and healthcare systems can only be mastered if the healthspan is also significantly extended. Adult stem cells hold great promise to achieving this goal, as they are key to ensuring lifelong organ and tissue function. Counteracting functional decline or depletion of stem cells is therefore a vital strategy to promote healthy aging. Past research has provided comprehensive insights into the molecular mechanisms that cause stem cell impairment with age and has put their epigenome at center stage. In fact, the plasticity of stem cell chromatin facilitates the production of a vast amount of progeny, but also makes the chromatin prone to epimutations. Although many issues are still to be unraveled, the recent advance in understanding the extrinsic and intrinsic factors causing this epigenetic erosion will facilitate the development of interventions to prevent, or even revert, stem cell aging.

## Figures and Tables

**Table 1:** Epigenetic differences between quiescent and activated MuSCs, and between young and old MuSCs. For details please see text.

	Young MuSCs	Old MuSCs
**Quiescent** **MuSCs**	H3K4me3 ● H3K27me3 ● bivalency ● H4K20me3 ↑ H4K16ac ↓	H3K4me3 ↓ H3K27me3 ↑ (at histone genes & globally) H4K16ac ↑
**Activated** **MuSCs**	H3K4me3 ● H3K27me3 ↑ bivalency ↑ H4K20me3 ↓ H4K16ac ↑	H3K4me3 ↑↑ (at Hoxa9 locus) H3K27me 3● H4K16ac ↑↑

● initial level or unchanged; ↑ up-regulated; ↑↑ strongly up-regulated; ↓ down-regulated.
